# Pulmonary Aspergilloma in a Non-adherent Systemic Lupus Erythematosus Patient Receiving Long-Term Immunosuppression: A Report of a Rare Case

**DOI:** 10.7759/cureus.88925

**Published:** 2025-07-28

**Authors:** Ariful Islam, Sharmin Nahar, Md. Abu Shahin, Syed Jamil Abdal

**Affiliations:** 1 Rheumatology, Bangladesh Medical University, Dhaka, BGD

**Keywords:** autoimmune disease, bronchial artery embolization, immunosuppression, pulmonary aspergilloma, systemic lupus erythematosus (sle), systemic sclerosis (ssc), voriconazole therapy

## Abstract

Pulmonary aspergilloma is an uncommon but potentially life-threatening condition that predominantly affects individuals with pre-existing structural lung disease and immunosuppression. Systemic lupus erythematosus (SLE), especially with long-term immunosuppressive therapy, significantly increases susceptibility to opportunistic infections, including fungal pathogens such as *Aspergillus* species. These patients are also at heightened risk for a broad range of opportunistic infections, such as *Candida* species, viral infections such as herpes zoster and cytomegalovirus (CMV), *Mycobacterium tuberculosis*, and *Pneumocystis jirovecii* pneumonia (PJP). We report a case of a 50-year-old woman with long-standing SLE and poorly controlled diabetes mellitus who developed multilobar pulmonary aspergilloma while on chronic glucocorticoids and azathioprine. Despite initial improvement, she developed hemoptysis, necessitating bronchial artery embolization consideration. Due to extensive bilateral disease, surgical intervention was deferred, and she was successfully managed with prolonged voriconazole therapy. This case underscores the diagnostic and therapeutic challenges of aspergilloma in immunocompromised hosts and highlights the importance of vigilant monitoring and tailored antifungal strategies.

## Introduction

Chronic pulmonary aspergillosis (CPA) encompasses a spectrum of diseases caused by *Aspergillus* species, a genus of environmental fungi (mold). These fungi can cause serious infections, particularly in immunocompromised individuals, with clinical manifestations including aspergilloma, chronic cavitary, and fibrosing pulmonary aspergillosis. Aspergilloma, also known as a fungal ball, forms within pre-existing pulmonary cavities, often resulting from tuberculosis, sarcoidosis, or other chronic pulmonary conditions [1,2]. Systemic lupus erythematosus (SLE) is a chronic autoimmune disease marked by immune-mediated damage to multiple organs. Immunocompromised patients, including those with systemic lupus erythematosus (SLE), are at heightened risk due to impaired immune surveillance and long-term immunosuppressive therapy [3,4].

While aspergilloma may remain asymptomatic, it can manifest with chronic cough, weight loss, and hemoptysis, which may be life-threatening. Diagnosis relies on imaging, microbiology, and serology, and treatment options vary based on disease extent, symptoms, and host immune status. Surgical resection is preferred in localized disease, whereas antifungal therapy and embolization are mainstays for multifocal or inoperable cases [5].

We present a rare case of multilobar pulmonary aspergilloma in a patient with SLE and poorly controlled diabetes mellitus, highlighting management challenges in this vulnerable population.

## Case presentation

A 50-year-old woman with a 26-year history of SLE presented with recurrent hemoptysis for 3-4 months and worsening shortness of breath over 15 days. Her background history included type 2 diabetes mellitus for the last 14 years. Initially, she was managed with linagliptin-metformin, and later on, gliclazide and empagliflozin were added, although glycemic control remained poor. Recently, antidiabetic medications were adjusted, and insulin was added for better control of her diabetes.

Her SLE diagnosis was based on photosensitivity, facial rash, alopecia, recurrent oral ulcers, and serologic criteria. Later, she developed Raynaud's phenomenon and digital ulcers, for which she was put on a calcium channel blocker and tadalafil. Her nailfold capillaroscopy revealed a nonspecific pattern. Her symptoms improved. However, she was lost to follow-up and had been maintained on azathioprine (50-100 mg/day) along with low-dose glucocorticoids (10 mg/day) for more than two decades.

**Figure 1:**
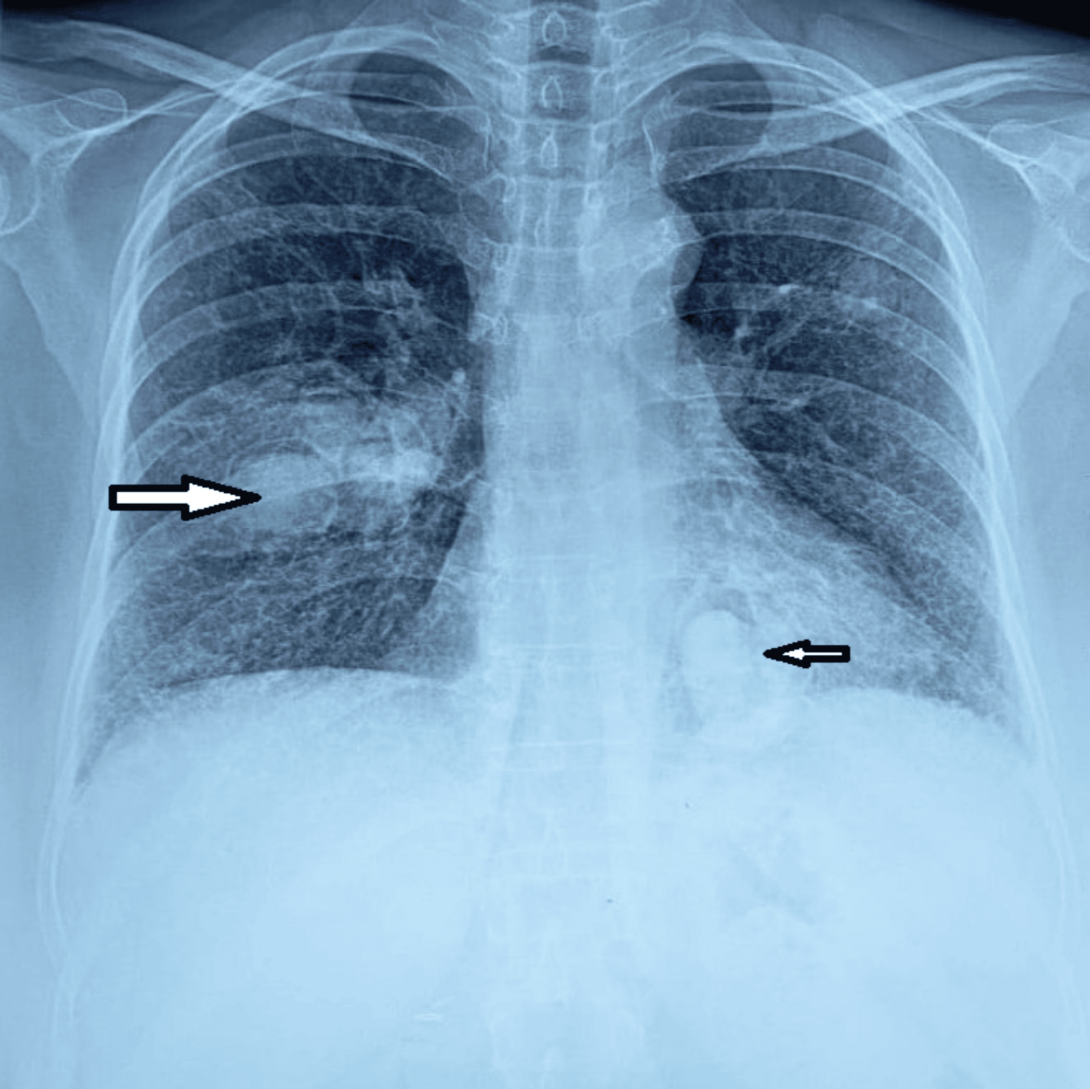
Chest X-ray posteroanterior view showing multiple aspergilloma with DPLD Multiple nodular opacities (aspergilloma) are noted in the right hilar (large arrow) and left lower zones (small arrow), all of them having a thin crescentic air lucency around them. Reticular opacities are noted in both lower zones. DPLD: diffuse parenchymal lung disease

For the past six years, she had progressive exertional dyspnea and dry cough, but no prior hemoptysis or fever. She was taking azathioprine and steroids until her recent symptoms appeared. On chest examination, breath sounds are mildly diminished in the right mid-lung zone, accompanied by bilateral basal crepitations. Echocardiography showed preserved ejection fraction (EF) (61%) with mildly elevated pulmonary arterial systolic pressure (PASP) (40 mmHg). Pulmonary function testing indicated moderate restriction with reduced diffusing capacity of the lungs for carbon monoxide (DLCO) and significant desaturation (peripheral oxygen saturation (SpO_2_): 82%) during the six-minute walk test. Her chest X-ray (Figure 1) shows multiple nodular lesions in both lung fields, having a thin crescentic air lucency and reticular opacity in the lower zone.

**Figure 2:**
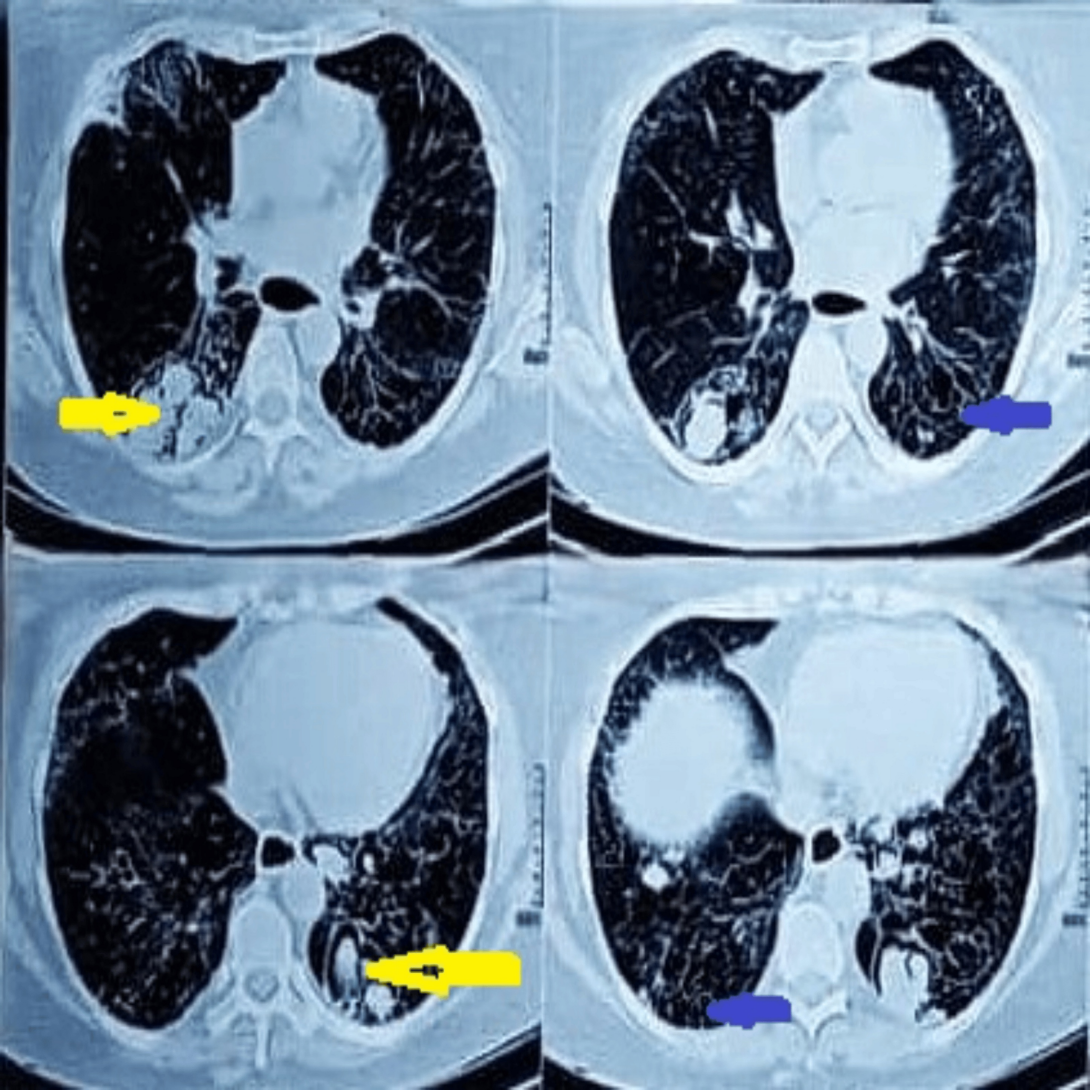
HRCT showing a cavitary lesion with a fungal ball (aspergilloma) on the background of DPLD Non-enhancing soft tissue density lesion (aspergilloma) with a peripheral crescentic margin, with associated air space (yellow arrows on right and left sides) densities noted on both lung fields, and interstitial thickening with honeycombing (blue arrow) are seen in both basal regions. HRCT: high-resolution computed tomography, DPLD: diffuse parenchymal lung disease

Subsequent computed tomography (CT) imaging (Figure 2) revealed multiple bilateral thin-walled cavitary lesions containing fungal balls with associated air-crescent signs, findings consistent with pulmonary aspergilloma on the background of diffuse parenchymal lung disease (DPLD).

The initial differential included pulmonary tuberculosis due to endemicity and immunosuppression; however, sputum analysis, including Gram stain, acid-fast bacillus (AFB) staining, culture and sensitivity, and repeated AFB smears and cultures, was negative. Giemsa stain demonstrated fungal elements, and serum galactomannan and 1,3-β-D-glucan were elevated, supporting a diagnosis of chronic pulmonary aspergillosis. Table 1 represents the laboratory data.

**Table 1:** Laboratory investigations CBC: complete blood count, Hb: hemoglobin, MCV: mean corpuscular volume, MCH: mean corpuscular hemoglobin, RBC: red blood cell count, WBC: white blood cell count, ESR: erythrocyte sedimentation rate, CRP: C-reactive protein, SGPT: serum glutamic pyruvic transaminase, UTP: urinary total protein, FBS: fasting blood sugar, ANA: antinuclear antibody, dsDNA: double-stranded DNA, C-ANCA: cytoplasmic antineutrophil cytoplasmic antibody, P-ANCA: perinuclear antineutrophil cytoplasmic antibody, ELISA: enzyme-linked immunoassay

Investigation	Result	Reference range
CBC		
Hb	12.1	13.5±1.3
MCV	75.8 fL	92±9 fL
MCH	23 pg	29.5±2.5 pg
RBC	5.25×10^12^/L	4.3±0.5×10^12^/L
WBC	9.5×10^9^/L	7±3×10^9^/L
Platelet	500×10^9^/L	150-450×10^9^/L
ESR	50 mm in the first hour	0-10 mm in the first hour
CRP	12.8 mg/L	<6 mg/L
SGPT	13 U/L	Up to 59 U/L
24-hour UTP	0.13 gm/day	<0.2 gm/day
Serum creatinine	0.8 mg/dL	0.6-1.3 mg/dL
Hemoglobin A1c	10.4%	4.9%-6%
FBS	9.2 mmol/L	3.5-6 mmol/L
Galactomannan antigen	2.64	>0.5 (positive)
1,3 β-D-glucan	124.16 pg/mL	<80 (low risk), >100 (high risk)
Serological		
ANA	Moderately positive	-
Anti-dsDNA antibody	40	Cutoff rate: 30
C-ANCA	2 U/mL	<5 U/mL (negative) (ELISA)
P-ANCA	2.1 U/mL	<5 U/mL (negative) (ELISA)
Anti-Scl-70	9.69 U/mL	<20 U/mL (negative)
Anti-Centromere Ab	<0.5	<20 (negative)

Laboratory investigations revealed normocytic anemia, elevated erythrocyte sedimentation rate (ESR) and C-reactive protein (CRP) levels, with stable liver, urine R/E, and renal function. Antinuclear antibody (ANA) (coarse speckled pattern) and anti-double-stranded DNA (anti-dsDNA) (low titer) were positive, while antineutrophil cytoplasmic antibodies (ANCAs), anti-Scl-70, and anticentromere antibodies were negative. Antinuclear antibody (ANA) testing was performed using the indirect immunofluorescence (IIF) method on HEp-2 cells, while antineutrophil cytoplasmic antibodies (ANCAs), anti-Scl-70, and anticentromere antibodies were assessed using enzyme-linked immunosorbent assay (ELISA).

Following diagnosis, the patient shows no clinical or serological evidence of an SLE flare. However, she has concomitant pulmonary aspergilloma. In this context, azathioprine was discontinued, hydroxychloroquine was initiated, and corticosteroids were tapered gradually. Given the usual interstitial pneumonia (UIP) pattern on HRCT, antifibrotic therapy with nintedanib may be considered for her interstitial lung disease (ILD). However, there have been reports of opportunistic fungal infections, including talaromycosis, in patients receiving nintedanib. Therefore, the initiation of antifibrotic therapy is deferred until the current fungal infection is adequately treated and controlled. The patient was started on voriconazole 200 mg twice daily. While respiratory symptoms improved over four months, she developed low-volume hemoptysis (10-20 mL/day). Bronchial artery embolization was considered, but the procedure was deferred due to extensive bilateral cavitary disease. The patient continued voriconazole therapy with monthly monitoring of serum glutamic pyruvic transaminase (SGPT) initially. After stabilization, follow-up was planned at three-month intervals. She was regularly assessed for visual disturbances and neurological symptoms such as dizziness and headache, but no such symptoms developed. Additionally, an electrocardiogram (ECG) was performed to assess for any abnormalities. SGPT levels remained within normal limits throughout the follow-up period.

After seven months of antifungal treatment, hemoptysis resolved completely, and she has remained symptom-free for the past three months. We are planning to continue antifungal treatment for a total duration of one year, provided there is no recurrence of symptoms or radiological evidence of disease progression.

## Discussion

Aspergilloma typically develops in pre-existing pulmonary cavities, with *Aspergillus fumigatus* being the most common species implicated. Immunosuppression, especially from long-term corticosteroids or cytotoxic agents, is a known risk factor [6]. Patients with autoimmune diseases such as SLE often require prolonged immunosuppression, placing them at high risk for opportunistic fungal infections [7,8].

Our patient comes from a middle-class background. Although she responded relatively well to azathioprine and steroid therapy, treatment non-adherence remains a significant concern. Contributing factors include the long distance to the healthcare facility, limited understanding of her illness, inadequate follow-up, and insufficient counseling and support. These barriers highlight the multifaceted nature of treatment non-adherence, which can significantly impact disease control and long-term outcomes.

Interstitial lung disease (ILD) is an uncommon manifestation of systemic lupus erythematosus (SLE), with a reported prevalence ranging from 3% to 13% in small clinical studies [9,10] and approximately 2% in the large RELESSER TRANS registry [11], which included 3,215 SLE patients. The prevalence of ILD in SLE appears to vary geographically [12]. Notably, ILD occurs more frequently in SLE patients with coexisting autoimmune conditions such as Sjögren's syndrome or systemic sclerosis [13]. However, in our case, there were no clinical signs or serological evidence suggestive of an overlap syndrome.

In our patient, multiple risk factors converged: chronic lung changes from ILD, long-term azathioprine and steroid therapy, and poorly controlled diabetes. The diagnosis of aspergilloma was supported by radiological findings (air-crescent sign and cavitary lesions with fungal balls) and serologic markers (elevated galactomannan and β-D-glucan). Microbiological confirmation was aided by Giemsa staining.

Treatment of aspergilloma depends on symptoms, extent of disease, and surgical feasibility. Surgical resection is typically recommended for localized disease with significant hemoptysis [14]. However, in patients with multilobar disease, poor pulmonary reserve, or immunocompromised states, medical therapy becomes the primary approach [15].

Voriconazole is the first-line antifungal agent for CPA and has shown efficacy in improving symptoms and stabilizing radiological findings. In this case, prolonged therapy (≥7 months) led to the resolution of hemoptysis and sustained clinical stability [16]. Based on a multicenter trial [16] recommending voriconazole (200 mg twice daily) for 6-12 months in minimally or non-immunocompromised patients, we plan to extend the treatment to 12 months, considering her immunosuppressed status. The deferral of bronchial artery embolization and surgery highlights the importance of individualized, conservative approaches in complex patients.

This case illustrates the need for heightened awareness of CPA in immunocompromised hosts with chronic lung symptoms and the value of integrating imaging, serology, and mycology in diagnosis. Long-term follow-up is essential due to the risk of relapse or complications.

## Conclusions

This case highlights the complexity of managing opportunistic infections such as pulmonary aspergilloma in patients with connective tissue disease, particularly when complicated by chronic immunosuppression and poor follow-up. SLE-associated interstitial lung disease, long-standing corticosteroid and immunosuppressive use, and poorly controlled diabetes created a high-risk milieu for fungal colonization and secondary infection. Clinicians should maintain a high index of suspicion for fungal etiologies in immunocompromised patients presenting with new or worsening respiratory symptoms, especially in tuberculosis-endemic regions. Early diagnosis through imaging and the use of fungal biomarkers, combined with the timely initiation of antifungal therapy, is critical for achieving optimal outcomes. This case underscores the importance of regular follow-up and comprehensive management of comorbidities in autoimmune disease patients.
